# Acute Presentation of Structural Valve Degeneration in a Transcatheter Heart Valve (Sapien XT) at 7.5 Years; Successful Redo TAVR With a Sapien 3 Ultra

**DOI:** 10.1016/j.cjco.2020.11.007

**Published:** 2020-11-17

**Authors:** Sagar N. Doshi, Adnan Nadir, William Moody, Jonathan N. Townend

**Affiliations:** Queen Elizabeth Hospital Birmingham, Edgbaston, Birmingham, United Kingdom

## Abstract

Little is known about the presentation of structural valve degeneration complicating transcatheter heart valves (THVs). We report a case of acute heart failure, secondary to leaflet prolapse, in a previously well 77-year-old man, 7.5 years after successful transcatheter aortic valve replacement with a 26-mm balloon-expandable Sapien XT (Edwards Lifesciences, Irvine, CA) THV. This case highlights that structural valve degeneration complicating THVs might lead to acute presentation with little warning from previous echocardiograms. Calcification might be absent on imaging. Redo transcatheter aortic valve replacement is feasible and appears safe. Post deployment optimization with a highly noncompliant balloon might improve full expansion of the newly implanted THV and improve valve performance.

## Résumé

On en sait peu sur les manifestations associées à une dégénérescence structurelle d'une prothèse valvulaire après implantation par cathéter. Nous présentons un cas d’insuffisance cardiaque aiguë secondaire à un prolapsus valvulaire chez un homme de 77 ans qui jusque-là allait bien, survenu 7,5 ans après l’implantation par cathéter d’une valve cardiaque de 26 mm Sapien XT (Edwards Lifesciences, Irvine, CA). Ce cas montre qu’une dégénérescence structurelle de la prothèse valvulaire peut entraîner une manifestation aiguë sans que des signes précurseurs soient détectés à l’échocardiographie. Il peut arriver que les examens d’imagerie ne révèlent pas de calcification. Il est possible d’implanter une autre valve aortique par cathéter pour remplacer la prothèse défaillante, une intervention qui semble sûre. Une optimisation à l’aide d’un ballonnet non compliant pourrait faciliter l’expansion complète de la nouvelle valve cardiaque implantée par cathéter et en améliorer le fonctionnement.

Until recently transcatheter aortic valve replacement (TAVR) has been used principally in elderly, high-risk populations with limited life expectancy. Failure of transcatheter heart valves (THVs) due to structural valve degeneration (SVD), requiring urgent reintervention, is an uncommon occurrence. There are relatively few data on the timing, mode of presentation, valvular imaging characteristics, and outcomes of redo TAVR in this situation. We describe the first case of redo TAVR for SVD at our institution after more than 1000 TAVR cases, 7.5 years after successful TAVR at our centre.

## Case

In March 2020, during the COVID-19 pandemic, a 77-year-old man was admitted emergently with a 5-month history of progressive exertional breathlessness (New York Heart Association [NYHA] classification III) and orthopnea. Before this he had been well and unlimited in his daily activity (NYHA class I). He denied fevers or sweats. Examination revealed evidence of heart failure with raised jugular venous pressure, mild chest crepitations, and ankle swelling. Auscultation revealed an ejection systolic murmur at the aortic area and an early diastolic murmur of aortic regurgitation. His C-reactive protein was elevated at 37 mg/L (reference < 5 mg/L), hemoglobin was low at 11.7 g/dL, and estimated glomerular filtration rate reduced at 43 mL/min. Two reverse transcriptase polymerase chain reaction assays on nasopharyngeal swabs were negative for SARS-CoV-2 virus.

**Figure 1:**
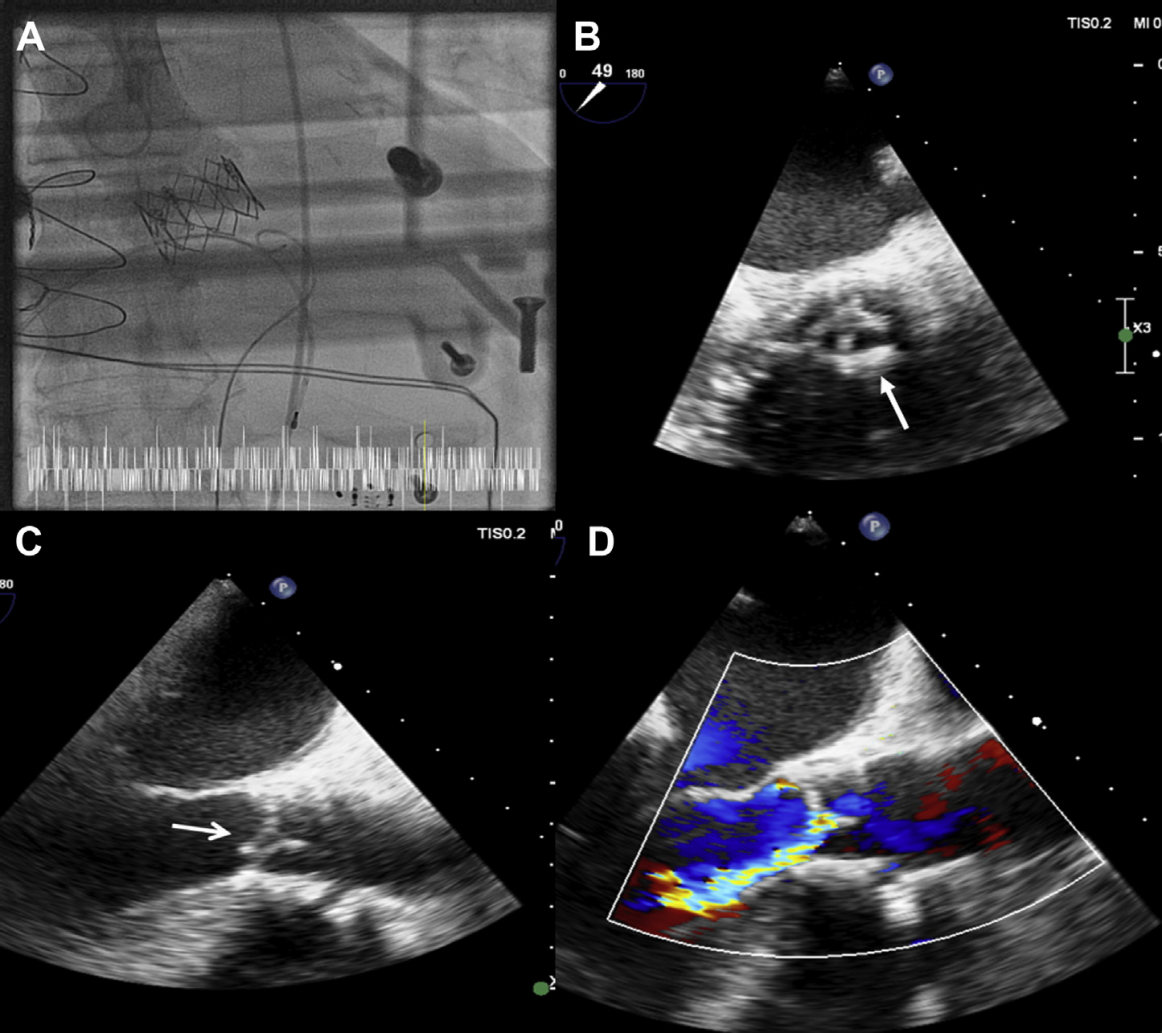
(**A**) Aortogram after deployment of 26-mm Sapien XT (Edwards Lifesciences Corp, Irvine, CA) valve in September 2012 for severe aortic stenosis showed absence of aortic regurgitation. (**B**) Transoesophageal echocardiogram (TEE) (March 2020), in the short axis view, showed good opening of the 26-mm Sapien XT valve but evidence of modest leaflet thickening, secondary to structural valve degeneration (**arrow**). (**C**) TEE (March 2020) in long axis view showed prolapse of a transcatheter heart valve cusp (**arrow**). (**D**) TEE (March 2020) in long axis view showed a jet of eccentric, severe aortic regurgitation (3D vena contracta area 0.31 cm^2^) due to cusp prolapse.

In September 2012 the patient underwent transfemoral TAVR with a 26-mm Sapien XT valve (Edwards Lifesciences, Irvine, CA) for severe aortic stenosis (Fig. 1A; see Video 1 ; view video online). At the time, the patient was 69 years of age and deemed high risk for open heart surgery because of previous bypass surgery in 2003, with 3 patent grafts, and acute presentation with kidney failure. After TAVR transthoracic echocardiogram (TTE) showed a peak velocity (VMax) of 1.6 m/s, mean gradient of 9 mm Hg, and aortic valve area (AVA) 2.02 cm^2^ with trivial paravalvular regurgitation and no transvalvular regurgitation. His left ventricular ejection fraction (LVEF) was 50%-55%. His other history included paroxysmal atrial fibrillation, mild ischemic stroke, and pacemaker implanted for symptomatic bradycardia.

In July 2019 the patient was investigated for atypical chest pain. TTE at that time showed an LVEF of 55% and a well functioning THV prosthesis. His VMax was 2.6 m/s, mean gradient 17 mm Hg, and AVA 1.5 cm^2^. There was no transvalvular regurgitation and trivial paravalvular regurgitation. Computed tomography (CT) coronary angiography showed 3 patent grafts.

**Figure 2:**
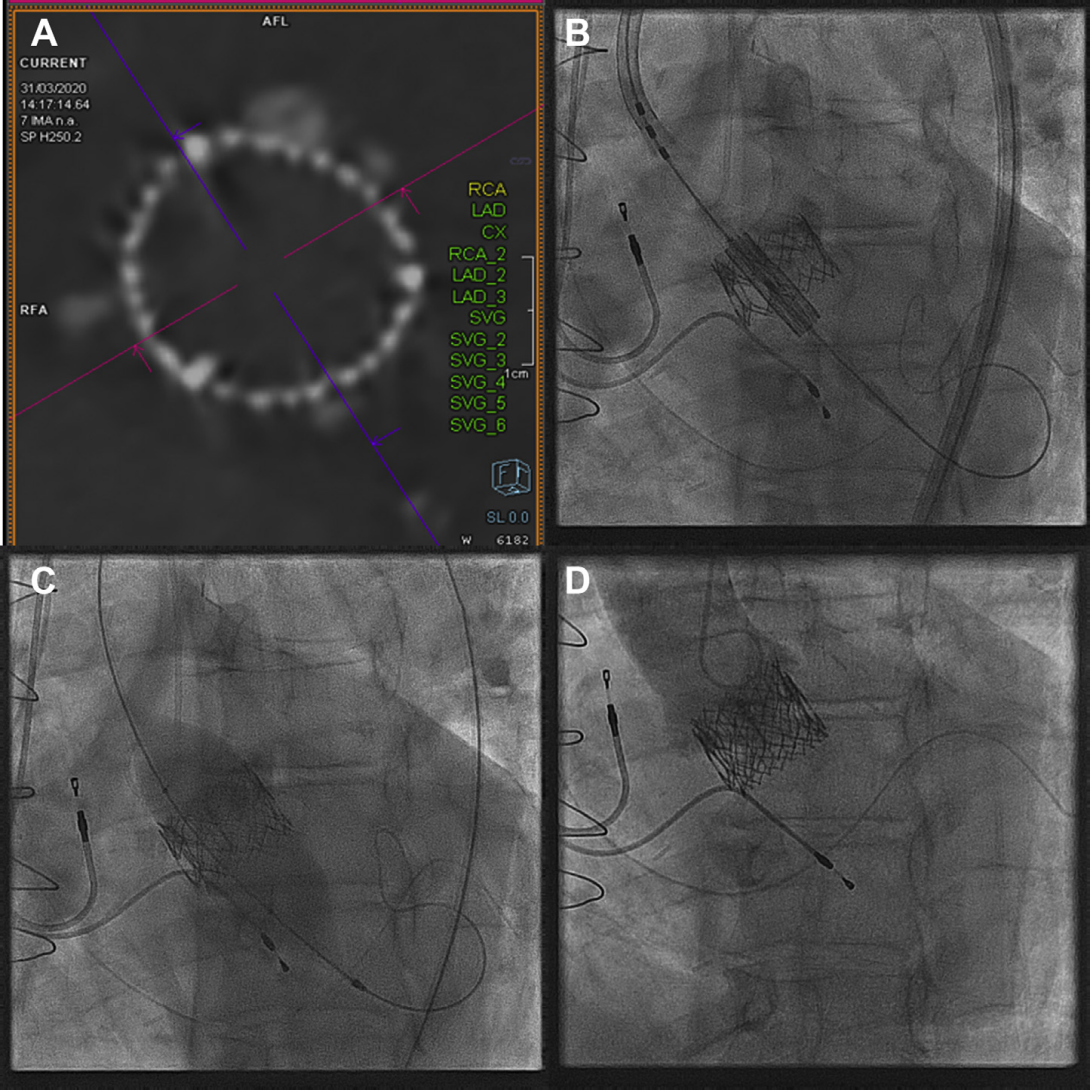
(**A**) Gated computed tomography aortogram (March 2020) showed complete absence of leaflet calcification of the Sapien XT (Edwards Lifesciences Corp, Irvine, CA) prosthesis (short axis left image, midframe level). (**B**) In April 2020 redo transcatheter aortic valve replacement was undertaken with a 26 mm Sapien 3 Ultra valve (Edwards Lifesciences Corp) via percutaneous transfemoral access. The top of the Sapien 3 Ultra was aligned with the top of the Sapien XT transcatheter heart valve. (**C**) Post dilatation was undertaken with a 26-mm Atlas Gold PTA Dilatation Catheter (Bard Peripheral Vascular, Inc, Tempe, AZ) balloon to 12 atm (nominal pressure 4 atm) to optimize expansion of the Sapien 3 and improve valve performance. (**D**) Root aortography after deployment and after optimization of the 26-mm Sapien 3 Ultra valve showed no aortic regurgitation.

The patient’s current presentation was believed to be consistent with a diagnosis of either infective endocarditis or SVD causing leaflet prolapse. Urgent TTE showed severe aortic regurgitation but could not discern if this was trans- or paravalvular. His VMax was 2.9 m/s, mean gradient 16 mm Hg, and left ventricular function was severely impaired (LVEF 30%). There was mild mitral regurgitation. Urgent transoesophageal echocardiogram (TEE) showed degenerative changes of the THV with leaflet thickening but good opening and no vegetations (Fig. 1B; see Video 2 ; view video online). There was a severe, eccentric, jet of aortic regurgitation (3D vena contracta area 0.31 cm^2^) due to prolapse of a THV cusp, (Fig. 1C and D; see Video 3  and Video 4 ; view videos online). A gated cardiac CT image showed no calcification of the THV leaflets, pannus formation, leaflet thrombus, or abscess (Fig. 2A; see Video 5 ; view video online).

Heart failure was treated with intravenous diuretics, angiotensin-converting enzyme inhibitors and β-blockers. Over 2 weeks, multiple blood cultures showed no bacterial growth. His C-reactive protein normalized without antibiotic therapy and the patient’s breathlessness improved. SVD was now believed to be the most likely diagnosis. The patient was deemed high risk for open heart surgery and percutaneous transfemoral valve in valve TAVR was considered the optimal treatment. The pacemaker was upgraded to a CRT-P (Boston Scientific, Marlborough, MA) and in April 2020, the patient underwent transfemoral TAVR with a 26-mm Sapien 3 Ultra (Edwards Lifesciences Corp) valve. The native coronary arteries took origin well above the Sapien XT prosthesis and there was therefore deemed to be no risk of native coronary artery occlusion or impaired coronary access. The Sapien 3 Ultra was positioned to align the top of the frame with the top of the Sapien XT THV (Fig. 2B; see Video 6 ; view video online). Post dilatation was performed with a 26-mm Atlas Gold balloon (Bard Peripheral Vascular Inc, Tempe, AZ) to 12 atm (Fig. 2C). There was no aortic regurgitation on aortography (Fig. 2D; see Video 7 ; view video online). Post procedure TTE showed good function of the prosthesis with a VMax of 1.8 m/s, maximum pressure gradient of 13 mm Hg, mean pressure gradient of 6.7 mm Hg, and AVA 2.0 cm^2^ with no transvalvular or paravalvular regurgitation.

There were no complications and the patient was discharged the next day with Apixaban 5 mg oral anticoagulation twice daily and heart failure medications. At the 8-week review the patient reported steadily improving exercise capacity (NYHA class II) and freedom from orthopnea and ankle swelling. At 21 weeks the patient had improved further (NYHA class I) and had undergone no admissions. An echocardiogram showed improved left ventricular function (ejection fraction 43%), stable valve parameters, and no trans- or paravalvular regurgitation.

## Discussion

Although the durability of THVs has been shown to be good in high-risk cohorts, SVD can be expected to occur during extended follow-up, resulting in THV failure that might require reintervention.^1^, ^2^, ^3^ SVD might present as stenosis or regurgitation.^4^ In the largest observational study of redo TAVR, among 63,876 TAVR procedures, 212 patients underwent redo TAVR (0.22%).^5^ The median time to redo TAVR was 5 years. The indication for redo TAVR beyond 1 year was regurgitation or combined stenosis/regurgitation in 62.3%. The low rate of redo TAVR likely reflects the good durability of THVs and limited life expectancy of the cohort studied. In the 5-year follow-up of the randomized **P**lacement of **A**o**r**tic **T**ra**n**scathet**er** Valves (PARTNER) 2 study, in which TAVR was compared with open heart surgery in intermediate-risk patients (Society of Thoracic Surgeons 4%-8%), aortic valve interventions were more frequently observed in the TAVR group (3.2% vs 0.8%).^6^ Of the 21 reinterventions after TAVR, 10 were for progressive aortic stenosis whereas 11 were for aortic regurgitation, and 17 were treated with redo TAVR (1.7%).

In our case, THV failure appears to have occurred relatively insidiously. Although the TTE in July 2019 showed increased transvalvular velocities, compared with the immediate post-deployment TTE in 2012, there was no transvalvular regurgitation. The TEE of March 2020 showed modest leaflet thickening with good opening but severe aortic regurgitation due to leaflet prolapse. The CT aortogram showed absence of leaflet calcification. We postulate that a leaflet tear occurred as a result of SVD, resulting in leaflet prolapse with resultant severe aortic regurgitation.

Redo TAVR would appear to have a safety profile similar to first-time TAVR. There were no mortalities in patients who underwent redo TAVR in the 5-year PARTNER 2 follow-up study or the registry of 212 patients who underwent redo TAVR.^5^^,^^6^ However, a recently highlighted problem after redo TAVR is impaired access to the native coronary arteries due to displaced leaflets of the first THV. A recent study in 137 patients showed coronary angiography to be unfeasible in 31% after redo TAVR.^7^ This difficulty can be overcome by deeper placement of the initial THV into the left ventricular outflow tract but might be associated with more pacemaker requirement. In the study of redo TAVR in 212 patients, high residual gradients (mean > 20 mm Hg) were observed in 14.3%.^5^ In the present case, post dilatation was performed with a highly noncompliant balloon to optimize expansion of the THV with the aim of improving valvular performance.

The peak and mean gradients observed after balloon optimization in our case were similar to the parameters observed after the first TAVR in 2012. This observation would support the notion that final dilatation with a highly noncompliant balloon might be reasonable, in an attempt to optimize expansion of the newly implanted THV and thus improve valve performance.

**Novel Teaching Points**

• This case highlights that SVD complicating THVs might lead to acute presentation with heart failure with little warning from previous echocardiograms.
• Unlike degeneration in native aortic valves, calcification might be absent.
• Redo TAVR is feasible and appears to have a safety profile similar to first-time TAVR.
• Post deployment optimization with a highly noncompliant balloon might serve to facilitate full expansion of the newly implanted THV and improve valve performance.

## Funding Sources

The authors have no funding sources to declare.

## Disclosures

Dr Doshi is a proctor for Edwards Lifesciences. The other authors have no conflicts of interest to disclose.

## Supplementary Material

Video 1 Aortogram post 26-mm Sapien XT (Edwards Lifesciences Corp, Irvine, CA) deployment September 2012.

Video 2 TEE short axis view showing thickening of the Sapien XT (Edwards Lifesciences Corp, Irvine, CA) leaflets and good opening.

Video 3 TEE long axis showing prolapse of the transcatheter heart valve leaflet.

Video 4 TEE long axis with colour showing eccentric jet of severe aortic regurgitation.

Video 5 Short axis run of gated computed tomography imaging through the transcatheter heart valve showing absence of calcification.

Video 6 Valve in valve transcatheter aortic valve replacement with 26-mm Sapien 3 Ultra transcatheter heart valve (Edwards Lifesciences Corp, Irvine, CA).

Video 7 Aortogram after deployment of 26-mm Sapien 3 Ultra transcatheter heart valve (Edwards Lifesciences Corp, Irvine, CA).
